# Low ALT Levels Associated with Poor Outcomes in 8700 Hospitalized Heart Failure Patients

**DOI:** 10.3390/jcm9103185

**Published:** 2020-09-30

**Authors:** Amitai Segev, Edward Itelman, Chen Avaky, Liat Negru, Gilat Shenhav-Saltzman, Avishay Grupper, Yishay Wasserstrum, Gad Segal

**Affiliations:** 1 Internal Medicine “T”, Chaim Sheba Medical Center, Sackler Faculty of Medicine, Tel-Aviv University, Ramat-Gan 5266202, Israel; agsegev@yahoo.com (A.S.); edi.itelman@gmail.com (E.I.); chenavaky@gmail.com (C.A.); liatneg@gmail.com (L.N.); gilatshenhav@gmail.com (G.S.-S.); yishay.wasserstrum@gmail.com (Y.W.); 2 Cardiovascular Division, Chaim Sheba Medical Center, Sackler Faculty of Medicine, Tel-Aviv University, Ramat-Gan 5266202, Israel; avishay.grupper@sheba.health.gov.il

**Keywords:** heart failure, sarcopenia, frailty, ALT, prognosis

## Abstract

Sarcopenia and frailty are causes for morbidity and mortality amongst heart failure (HF) patients. Low alanine transaminase (ALT) is a marker for these syndromes and, therefore, could serve as a biomarker for the prognostication of HF patients. We performed a retrospective analysis of all consecutive hospitalized HF patients in our institute in order to find out whether low ALT values would be a biomarker for poor outcomes. Our cohort included 11,102 patients, 35.6% categorized as heart failure with reduced ejection fraction. We excluded patients with ALT > 40 IU/L and cirrhosis. 8700 patients were followed for a median duration of 22 months and included in a univariate analysis. Patients with ALT < 10 IU/L were older (mean age 78.6 vs. 81.8, *p* < 0.001), had past stroke (24.6% vs. 19.6%, *p* < 0.001), dementia (7.7% vs. 4.6%, *p* < 0.001), and malignancy (13.4% vs. 10.2%, *p* = 0.003). Hospitalization length was longer in the low-ALT group (4 vs. 3 days, *p* < 0.001), and the rate of acute kidney injury during hospitalization was higher (19.1% vs. 15.6%; *p* = 0.006). The in-hospital mortality rate was higher in the low-ALT group (6.5% vs. 3.9%; *p* < 0.001). Long-term mortality was also higher (73.3% vs. 61.5%; *p* < 0.001). In a multivariate regression analysis, ALT < 10 IU/L had a 1.22 hazard ratio for mortality throughout the follow-up period (CI = 1.09–1.36; *p* < 0.001). Low ALT plasma level, a biomarker for sarcopenia and frailty, can assist clinicians in prognostic stratification of heart failure patients.

## 1. Introduction

Heart failure (HF), either with reduced ejection fraction (HFrEF) or preserved ejection fraction (HFpEF), remains a rising global epidemic and a major cause of re-hospitalizations, increased overall (both short- and long-term) mortality, limited exercise capacity, and significantly lower quality of life [1,2,3,4]. Frailty is a clinical syndrome manifesting as increased vulnerability from age-related decline in physiological reserve and function, leading to a reduced ability to tolerate biological stressors. Operationally, Fried et al. defined frailty as fulfilling three or more of the following criteria of reduced gait speed, reduced grip strength, exhaustion, unintended loss of body weight, and lowered physical activity [5]. Frailty is the phenotypic entity resulting from sarcopenia. Both sarcopenia and frailty are increasingly being recognized as significant contributors of morbidity and mortality, not only amongst the most elderly but also in mid-life patients [6,7]. Approximately 25% of older patients with HF exhibit evidence of frailty [8,9], increasingly recognized as a well-established predictor of negative clinical outcomes in such patients [10,11,12,13]. A long-term search for available biomarkers of sarcopenia and frailty is still ongoing, both for the general population of frail patients and those with both frailty and heart failure. 

Alanine transaminase (ALT) serves in many tissues to facilitate the conversion of pyruvate to the amino acid alanine. As such, it helps to reuse potentially “dead-end” carbohydrates within skeletal muscle tissue, after re-conversion of alanine to pyruvate within the liver. The most common use for ALT measurements has long been for monitoring liver tissue damage. Nevertheless, when the liver parenchyma is intact, ALT blood levels (demonstrated as catalytic activity) are a good marker for the whole-body skeletal muscle mass [14,15]. Utilizing the aforementioned, a large body of evidence accumulated in the past years, marking lower ALT values as a reliable marker for sarcopenia and frailty, in large, heterogeneous patient populations [16,17,18,19]. Characterizing sarcopenia and frailty status in patients with HF may provide clinicians with an indicator for gauging disease severity, prognosis, and disease progression or reversal. The role of plasma ALT activity in HF patients has not been described thus far.

## 2. Materials and Methods

We performed a retrospective analysis of all consecutive patients admitted between 1/3/2007 and 31/1/2020 at Chaim Sheba Medical Center, the largest tertiary hospital in Israel, with a main diagnosis of HF or a related ICD-10 code. Patients or the public were not involved in the design, conduct, reporting, or dissemination plans of our research. Excluded from the study were patients with plasma ALT activity above normal (>40 IU/L) and patients with a diagnosis of cirrhosis at baseline, since it is assumed that their ALT blood level does not reflect the total muscle mass and might be derived from damaged liver tissue as well. Exclusion of such patients was the rule in all other, previous ALT-associated sarcopenia and frailty studies [14,17,20,21,22,23,24]. We retrieved patients’ data from their electronic medical records after study approval by the local institutional review board.

We collected all relevant medical history and background diagnoses from the coded electronic patient chart. Congestive-HF-related admissions were considered as readmissions with a main diagnosis of HF or other related ICD-10 codes, at our institution. Laboratory data were taken from the first available laboratory tests within the index hospitalization. We defined acute kidney injury (AKI) as an increase in serum creatinine by ≥0.3 mg/dL or an increase to ≥1.5 times the baseline value upon admission. Echocardiography data were retrieved from the most recent exam available within 6 months before or after the index hospitalization (90% of the exams were performed between 13 days prior to and 12 days after the index hospitalization). Mortality data were extracted from the Israeli National Population Register and were available for all cases. Patients were stratified into two groups based on their first ALT measurement within the index hospitalization: low-normal ALT group (ALT < 10 IU/L) vs. high-normal ALT group (ALT ≥ 10 IU/L). We adopted the cutoff value of 10 IU/L from previous research in this field [20,21,24]. We compared clinical, laboratory, and echocardiographic parameters between these groups.

### Statistical Analysis

We described variables according to their properties. Categorical variables are reported in frequencies and percentages, and significance was assessed using the chi-square test or Fischer’s exact test when appropriate. Continuous variables with a normal distribution were reported as mean and standard deviation values, and significance was assessed using the Student’s t-test. Continuous variables that did not have a normal distribution were reported as median and interquartile range (IQR, 25th–75th percentiles) values, and significance was assessed using the Mann–Whitney U test. All statistical tests were two-sided, and a *p*-value of less than 0.05 was considered significant. We constructed a Cox regression model with the following variables to control for properties contributing to mortality: age, gender, history of cerebrovascular disease (defined as previous stroke or transient ischemic attack), history of diabetes mellitus (DM), history of ischemic heart disease (IHD), hemoglobin levels, creatinine clearance (estimated by the MDRD formula), albumin levels, left ventricular ejection fraction (LVEF), and severe right ventricular (RV) dysfunction. We used follow-up time and death as the event recorded. We constructed a forest plot according to the above model results. We used a Kaplan–Meier curve for survival, with death as the accumulating event for a follow-up time of up to 5 years. The statistical analysis was carried out with the use of R version 3.6.1 software (The R Foundation) and PyCharm community edition, V2020.1.1 (JetBrains).

## 3. Results

### 3.1. Patient Characteristics

**Figure 1:**
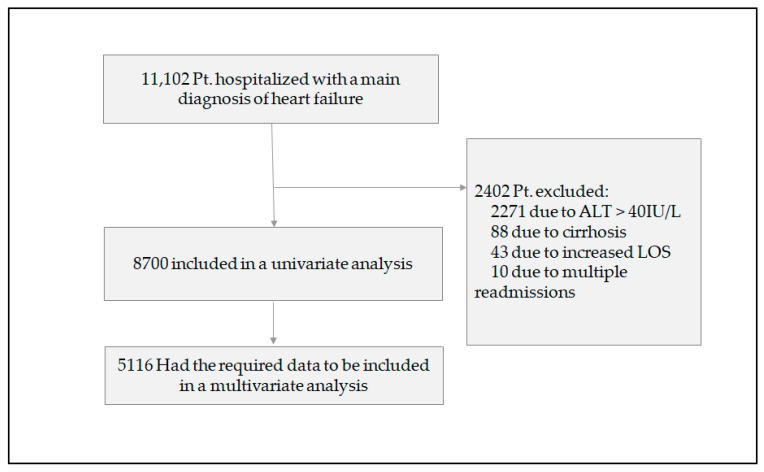
Flow and exclusions in the whole study cohort. LOS = length of (hospital) stay.

The study cohort consisted of 11,102 patients who were admitted at our medical center with a main diagnosis of HF exacerbation. Figure 1 shows the total patient cohort flow and exclusions. 8700 patients (55% males) were followed for a median duration of 22 (6–49) months and were eligible for inclusion in a univariate analysis. Out of these, 35.6% of patients were categorized as HFrEF. The cohort population manifests a high comorbidity rate (70% hypertension, 49% ischemic heart disease, 42% diabetes, 41% atrial fibrillation, 19% chronic kidney disease, 16% chronic obstructive pulmonary disease (COPD), and 14% solid or hematologic malignancy). Table 1 shows the patients’ characteristics according to their ALT activity level in the blood. Patients with ALT lower than 10 IU/L were older (mean age 78.6 vs. 81.8 years, *p* < 0.001), had a history of cerebrovascular disease (defined as previous stroke or transient ischemic attack; 24.6% vs. 19.6%, *p* < 0.001), suffered more often from dementia (7.7% vs. 4.6%, *p* < 0.001), and had a higher incidence of malignancy (13.4% vs. 10.2%, *p* = 0.003). Regarding baseline laboratory parameters, patients in the low-ALT group had lower hemoglobin concentration (10.6 vs. 11.5 g/dL, *p* < 0.001), worse renal function according to the MDRD values (43.2 vs. 51.5 mL/min/1.73 m^2^, *p* < 0.001) and a lower albumin concentration (3.4 vs. 3.6 g/dL, *p* < 0.001). HF patients with lower ALT values had better systolic cardiac function as reflected in their left ventricular ejection fraction (LVEF) values (55% vs. 50%, *p* = 0.006). Other echocardiographic parameters did not differ between the groups, including systolic pulmonary artery pressure (SPAP), the presence of mitral or tricuspid regurgitation, and right ventricular (RV) size and function.

### 3.2. Short- and Long-Term Outcomes

**Figure 2:**
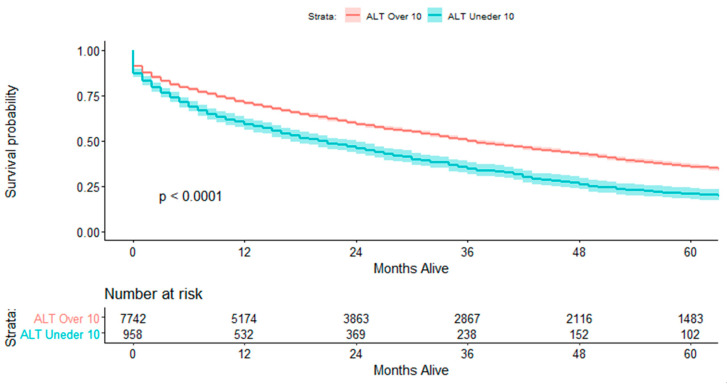
Survival according to baseline ALT level.

Hospitalization length was longer in the low-ALT group compared to that in the high-ALT group (4 vs. 3 days, *p* < 0.001), and the rate of acute kidney injury (AKI) was significantly higher in HF patients with low ALT values (19.1% vs. 15.6%; *p* = 0.006) (Table 2). Mortality rate within the index hospitalization was higher in the low-ALT group compared to that in the high-ALT group (6.5% vs. 3.9%; *p* < 0.001). Long-term mortality was also significantly higher in the low-ALT group (73.3% vs. 61.5%; *p* < 0.001) (Figure 2). Hospital readmission rate, both total and HF related, did not differ between the two groups.

### 3.3. Multivariate Analysis

**Figure 3:**
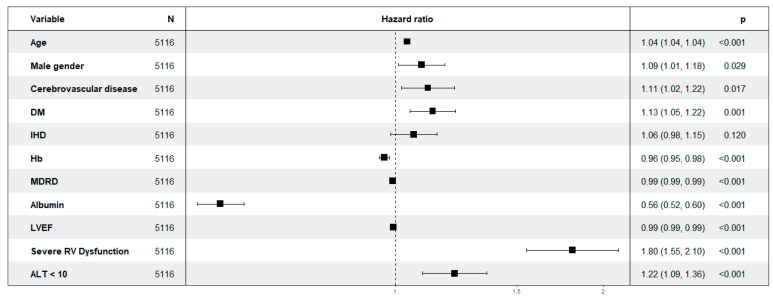
Forest plot depicting multivariate analysis for mortality.

We performed a multivariate regression model analysis showing that after factoring in clinically important parameters, ALT below 10 IU/L had a 1.22 hazard ratio (HR) for mortality throughout the follow-up period (confidence interval (CI) 1.09–1.36; *p* < 0.001) (Figure 3).

## 4. Discussion

This is the first study, thus far, to our knowledge, to evaluate the clinical utility of ALT plasma activity as an independent prognostic factor in hospitalized HF patients. In our study, low ALT plasma activity was associated with longer hospitalization stay, increased rates of kidney injury during the index hospitalization, and increased mortality, both in-hospital and long-term. A multivariate model further demonstrated the strong independent association between low ALT and mortality, sustained even after including other clinically important baseline comorbidities.

HF remains an important cause for morbidity and mortality, especially in the elderly. Whereas the prevalence of HF is approximately 1–2% of the adult population in developed countries, it rises to over 10% among people 70 years of age or older. The hospitalization and mortality rate in HF patients is extremely high. In the European ESC-HF pilot study, the 12-month rates in hospitalized HF patients were 44% and 17%, respectively [25]. 

Frailty is increasingly recognized as an important target for monitoring and intervention in contemporary cardiovascular care and management [26,27]. The role of frailty assessment in management decision-making and for the determination of procedural eligibility has been previously shown to affect patient outcomes and provide prognostic indication of well-being and survival associated with procedures, ranging from transcatheter and surgical aortic valve replacement to heart transplantation [27,28,29]. In HF patients, frailty is not only very common, reaching over 70% in patients 80 years of age or older [30], but also is an important prognostic factor. A 2018 systematic review of 14 studies including over 5000 chronic HF patients showed an association between frailty and increased risk for mortality (HR = 1.54; 95% CI 1.34–1.75; *p* < 0.001) and incident hospitalization (HR = 1.56; 95% CI 1.36–1.78; *p* < 0.001) [31]. Frailty is associated with circulating inflammatory cytokines and sarcopenia, features that are shared with HF [32]. DNA damage, impaired autophagy, and mitochondrial dysfunction occur in both aging and HF and can lead to metabolic dysfunction, cellular senescence, and ultimately cellular necrosis, in turn leading to the activation of innate immunity and production and secretion of inflammatory mediators into the circulation. The subsequent inflammation could, on the one hand, lead to frailty and could also negatively affect myocardial function via the negative inotropic effects of the circulating cytokines [33]. Systemic inflammation can also accelerate skeletal muscle apoptosis and promote sarcopenia [34], that could enhance immobility and cachexia associated with both HF and frailty.

The consensus regarding the importance of frailty assessment amongst HF patients only necessitates an available and reliable biomarker. Conventional assessment of frailty is done using frailty scoring systems, including walking speed (gait speed test), timed up-and-go test, PRISMA 7 questionnaire, Frail Score, Fried Score, and Short Physical Performance Battery (SPPB) [5,35,36].

Serum ALT is a readily available, inexpensive, and routine biochemical assay used in clinical practice. A large number of studies have established the association between low ALT levels and sarcopenia and frailty in both middle-aged [22] and elderly patients [16,37] and in a myriad of clinical contexts [18,19]. A prospective study of 179 patients in an internal ward found that low ALT blood activity and high Frail questionnaire score correlated with increased mortality and with each other [17]. Another study showed that an ALT > 10 IU/L among elderly patients with hip fracture was associated with better functional and cognitive status, as shown by higher total FIM (Functional Independence Measure) scores, cognitive FIM scores (>16), and FIM efficiency (>0.228) in a logistic regression analysis adjusted for age and gender (OR = 1.56–1.78) [24]. In clinical cardiology, low ALT levels were associated with increased long-term mortality among 6575 middle-aged patients with stable coronary heart disease [38], increased 90-day mortality in 6264 patients undergoing cardiovascular surgery [39], and lower baseline fitness and poor rehabilitation outcomes amongst 3806 participants of a cardiac rehabilitation program [23]. As opposed to the conventional assessment of frailty, ALT plasma measurement does not require additional substantial resources such as work force, money, and time.

In our study, we aimed to establish the association between low ALT levels and poor clinical outcomes in HF patients. To increase clinical utility, we selected the hospital admission as the point in time to evaluate the ALT level. In order to avoid potential bias after admission (e.g., prescribed medications affecting liver enzymes), we collected ALT levels upon admission. Abnormally high ALT levels (i.e., above 40 IU/L) might derive from damaged liver tissue and do not necessarily reflect the total body muscle mass and, therefore, cannot serve as a reliable marker for sarcopenia and frailty. Nevertheless, high ALT levels, as a marker of hypoperfusion and liver tissue damage, were previously found to be independently associated with increased mortality in HF patients [40].

In our study, patients in the low-ALT group had a higher rate of cerebrovascular disease, dementia, and malignancy at baseline. As these patients are less mobile, less robust, and more frail, they succumb to sarcopenia and are expected to have lower ALT blood levels. In addition, despite having higher LVEF at baseline, the low-ALT group demonstrated increased mortality. We believe that this observation further strengthens the association between low ALT and poor outcomes in HF patients, as manifested by better mortality prediction in a multivariate regression model (see Section 3.3. Multivariate Analysis).

Currently, frailty is not routinely assessed for or systematically categorized in patients with HF [41,42,43]. However, in our study, we found a strong association between low ALT levels and poor clinical outcomes, both short- and long-term, in a large cohort of hospitalized HF patients. This association, sustained after inclusion of other important prognostic markers, may be of great clinical importance.

### Limitations

We acknowledge some limitations to our study. First, this was an observational study with a retrospective analysis of collected data. Second, the study was conducted in a single tertiary medical center and there may have been patient selection bias. Thus, the data cannot necessarily be extrapolated to other centers. In addition, as in many studies, we relied on digitally encrypted events such as coded background diagnoses. Lastly, readmissions were considered in our center alone.

## 5. Conclusions

Our findings may significantly improve current clinical practices regarding heart failure patients. We believe the findings from our study should encourage the use of frailty assessment for risk stratification of patients with HF in order to inform prognosis and management decisions. We hereby suggest a novel, easy to measure, and cheap biomarker for frailty assessment in HF patients.

We believe that simple ALT measurement may be the first, small step, in the very long journey toward personalized medicine.

## Figures and Tables

**Table 1:** Patients’ characteristics according to their baseline alanine transaminase (ALT) blood activity level.

	ALT ≥ 10 IU/L (n = 7742)	ALT < 10 IU/L (n = 958)	Total (n = 8700)	*p*-Value
Patients’ demographics				
Age—years, median (IQR)	78.6 (69.4–85.6)	81.8 (72.7–87.5)	79 (69.8–85.8)	<0.001
Male gender—number (%)	4365 (56.4)	467 (48.7)	4832 (55.5)	<0.001
Comorbidities number (%)				
HTN	5358 (69.2)	686 (71.6)	6044 (69.5)	0.138
DM	3259 (42.1)	416 (43.4)	3675 (42.2)	0.453
Dyslipidemia	1842 (23.8)	200 (20.9)	2042 (23.5)	0.049
IHD	3789 (48.9)	445 (47.6)	4245 (48.8)	0.454
Cerebrovascular disease	1517 (19.6)	236 (24.6)	1753 (20.1)	<0.001
Atrial fibrillation	3237 (41.8)	376 (39.2)	3613 (41.5)	0.138
Dementia	354 (4.6)	74 (7.7)	428 (4.9)	<0.001
COPD	1272 (16.4)	170 (17.7)	1442 (16.6)	0.324
Solid malignancy	786 (10.2)	128 (13.4)	914 (10.5)	0.003
Hematologic malignancy	256 (3.3)	32 (3.3)	288 (3.3)	>0.99
CKD	1446 (18.7)	197 (20.6)	1643 (18.9)	0.173
Baseline laboratory parameters median (IQR)				
ALT (IU/L)	18 (14–25)	8 (7–9)	17 (12–24)	<0.001
Hemoglobin (g/dL)	11.5 (10.1–12.9)	10.6 (9.3–11.8)	11.4 (10–12.8)	<0.001
Creatinine (mg/dL)	1.2 (0.9–1.6)	1.3 (1–1.9)	1.2 (0.9–1.6)	<0.001
MDRD(mL/min/1.73 m²)	51.5 (35.7–69.1)	43.2 (28.8–63.4)	50.6 (34.8–68.5)	<0.001
Albumin(g/dL)	3.6 (3.3–3.9)	3.4 (3.1–3.7)	3.6 (3.2–3.9)	<0.001
Echocardiography findings				
LVEF %, median (IQR)	50 (30–60)	55 (35–60)	50 (30–60)	0.006
MR—n (%)				0.503
None	437 (9.5)	47 (8.5)	484 (9.4)	
Moderate	508 (11.0)	48 (8.6)	556 (10.7)	
Severe	222 (4.8)	23 (4.1)	245 (4.7)	
TR—n (%)				0.421
None	260 (5.6)	36 (6.5)	296 (5.7)	
Moderate	553 (12.0)	66 (11.9)	619 (12.0)	
Severe	307 (6.6)	47 (8.5)	354 (6.8)	
RVF—n (%)				0.81
Normal	3420 (74.0)	421 (75.9)	3841 (74.2)	
Severely reduced	249 (5.4)	28 (5.0)	277 (5.4)	
Dilated RV	1028 (22.3)	130 (23.4)	1158 (22.4)	0.576

IQR = interquartile range; HTN = hypertension; DM = diabetes mellitus; IHD = ischemic heart disease; COPD = chronic obstructive pulmonary disease; CKD = chronic kidney disease; LVEF = left ventricular ejection fraction; SPAP = systolic pulmonary artery pressure; MR = mitral regurgitation; TR = tricuspid regurgitation; LVEDD = left ventricular end diastolic diameter; RVF = right ventricular function; HFrEF = heart failure with reduced ejection fraction. Values in bold are statistically significant.

**Table 2:** Short-and long-term outcomes.

Outcome	ALT ≥ 10IU/L (n = 7742)	ALT < 10IU/L (n = 958)	Total (n = 8700)	*p*-Value
LOS—days, median (IQR)	3 (1–6)	4 (2–7)	3 (1–6)	**<0.001**
AKI—n (%)	1205 (15.6)	183 (19.1)	1388 (16.0)	**0.006**
Maximal creatinine—mg/dL	1.3 (1–1.9)	1.5 (1.1–2.2)	1.3 (1.0–1.9)	**<0.001**
In-hospital mortality—n (%)	304 (3.9)	62 (6.5)	366 (4.2)	**<0.001**
Readmission count—median (IQR)	2 (0–4)	2 (0–4)	2 (0–4)	0.489
HF readmissions—median (IQR)	0 (0–2)	0 (0–1)	0 (0–2)	0.795
Total mortality—n (%)	4764 (61.5)	702 (73.3)	5466 (62.8)	**<0.001**

LOS = length of (hospital) stay; IQR = interquartile range; AKI = acute kidney injury; HF = heart failure. Values in bold are statistically significant.
